# The interplay of life satisfaction and cognitive reserve: implications for cognitive changes in old age

**DOI:** 10.1186/s12877-026-07391-0

**Published:** 2026-03-28

**Authors:** Sara Pegoraro, Roberta Daini, Amaia Calderón-Larrañaga, Serhiy Dekhtyar

**Affiliations:** 1 Department of Psychology, University of Milano-Bicocca, Milan, Italy; 2 Aging Research Center, Department of Neurobiology, Care Sciences and Society, Karolinska Institutet, Stockholm, Sweden; 3 IRCCS Fondazione Don Carlo Gnocchi ONLUS, Milan, Italy

**Keywords:** Psychological well-being, Active aging, Cognitive decline

## Abstract

**Background:**

Life satisfaction (LS), a widely used measure of psychological well-being, has been previously linked with cognitive decline, although sex-differences, as well as the interplay of LS and other established correlates of cognitive health – particularly cognitive reserve (CR) – remain unknown. Thus, we explored the sex-specific interaction between LS an CR for cognitive change over 15 years during aging.

**Method:**

For this population-based longitudinal cohort study, data from the Swedish National Study on Aging and Care in Kungsholmen (SNAC-K) were used. The sample included 1871 cognitively intact individuals aged 60 years or older, followed up through regular clinical, functional, and psychological assessment for up to 15 years. Life Satisfaction Index was administered at baseline. CR was a latent factor incorporating education, work complexity, social network and leisure activities. Changes in the Mini-Mental State Examination (MMSE) over 15 years indicated cognitive decline. Linear mixed models, adjusted for personality, chronic disease burden, and depressive symptoms examined cognitive trajectories. The interplay of LSI with CR and sex was assessed in stratified analyses.

**Results:**

In mutually-adjusted models, higher LSI was associated with slower cognitive decline in women (β*time = 0.30; *p* < 0.001), but not in men (β*time = 0.10; *p* = 0.17), whereas higher CR was predictive of slower decline among both sexes. Among women, high LSI was associated with cognitive preservation in both low- (β*time = 0.40, *p* < 0.01) and high CR substrata (β*time = 0.26, *p* < 0.001), whereas in men, the null association of LSI with cognitive change remained across CR strata.

**Conclusions:**

Among women, promoting life satisfaction could be beneficial to prevent cognitive decline, whereas in men, cognitive reserve enrichment could be more efficacious. Overall, our findings underscored the importance of accounting for sex-specific differences when examining diverse determinants of cognitive health in old age, as potential targets for more personalized intervention among older adults.

**Clinical trial number:**

not applicable.

**Supplementary Information:**

The online version contains supplementary material available at 10.1186/s12877-026-07391-0.

## Introduction

In recent decades, the conceptualization of healthy aging has gradually moved away from a biomedical perspective that emphasizes physical health and a disease-free condition. Adopting a biopsychosocial approach has allowed a more integrated and realistic understanding of aging without neglecting the importance of psychological and social well-being [1, 2]. It results in a perspective that, while recognizing the aging-related losses in the physical and cognitive domains, also emphasizes the role of historical and cultural context, social structural/institutional forces and individual psychological components, mirroring the dynamic interplay between individual and environmental factors in shaping progression to aging [3, 4]. Particularly, psychological well-being has gained prominence as one of the main contributors to the achievement of an active aging condition and its hypothesized role in health maintenance [5]. In this context, well-being, as a multifaceted concept, has been operationalized in different ways, based on the dichotomy between the hedonic component, indicating a subjective assessment of affective status, as a balance between negative and positive emotions, and the eudaimonic one, which refers to the achievement of meaningful life goals [6, 7]. Among them, the life satisfaction (LS) has been one of the most widely used hedonic dimensions, representing a cognitive and global evaluation of the quality of one’s life, integrating pleasant and unpleasant affects and the perceived realization of wants [8].

Individuals with higher life satisfaction have been shown to exhibit a slower decline in physical function [9], reduced accumulation of multimorbidity [10], longer disability-free survival [11], fewer unplanned hospital admissions [12] and a lower mortality risk [13]. Several studies investigating these associations have also identified sex-specific differences, although with heterogeneous findings. For instance, life satisfaction has been found to be associated with mortality in men, but not women [14, 15]; its protective role on functional decline has been reported to be stronger in women compared to men [9]; while reduced life satisfaction has been linked with the development of chronic diseases, particularly in women [16].

Greater life satisfaction has also been associated with a slower rate of cognitive decline and reduced risk of dementia [17, 18]. The mechanisms underlying the cognitive benefits of life satisfaction could involve biological, behavioural and social pathways [e.g., 19, 20]. For instance, research suggests that individuals with high levels of global life satisfaction tend to experience better affect (i.e., more positive mood states), less perceived stress and have lower cortisol levels, with significant implications on immune and metabolic functioning [21, 22]. These individuals are also more likely to engage in healthy lifestyle behaviours (e.g., a diet rich in fruits and vegetables) and in pleasant activities to a greater extent [23, 24], thus minimizing the impact of several modifiable dementia risk factors [25]. The use of adaptive coping styles and a higher socioeconomic status, enabling more material resources to buffer life stressors, may further contribute to explaining the beneficial association between LS and cognition [24].

Although several studies have demonstrated the existence of a positive relationship between LS and cognitive functioning in aging [19, 26], few have considered the interplay of life satisfaction and other established determinants of cognitive aging and dementia risk, namely cognitive reserve (CR) [27]. On the one hand, the two concepts could connect through pathways involving mental health: the association of LS with depression and anxiety in late life is well established [28], while CR has not only been recently associated with late-life depressive burden [29], but its protective effects on cognition have also been shown to depend on depressive symptoms [30]. Furthermore, when examining CR and LS, pathways involving personality and stress are possible too, as previous literature has suggested that CR’s effect on cognitive outcomes could depend on specific personality traits [31] and circulating cortisol phenotypes [32], and life satisfaction is known to be impacted by both stress [33] and personality traits [34]. Together, this suggests that CR and LS can have both independent and synergistic effects on cognitive endpoints, although this relationship is largely unknown, particularly from longitudinal and sex-specific perspectives.

The rationale for investigating sex-specific differences in the interaction between LSI and CR on cognitive functioning derives from findings in the literature related not only to differences between men and women in the impact of life satisfaction on health outcomes, but also to a diverse influence of various dementia protection factors between sexes [35, 36]; it is also worth noting sex-specific discrepancies in cognitive functioning trajectories in aging, which may reflect either different patterns of resilience or different aging susceptibility across brain areas and contribute to sex differences in age-related decline, also depending on the cognitive domain [37, 38]. Sex differences in mental health, stress, and personality are also relevant here, as they may affect the pathways through which LS and CR exert independent or joint effects on cognitive outcomes.

Overall, understanding how life satisfaction and cognitive reserve interact could reveal novel ways of promoting resilience or strengthening its cognitive benefits, which are tailored differently for men and women. This could be especially beneficial for multidimensional interventions, targeting both structural (e.g., cognitive training) and experiential (e.g., well-being) components, with the aim of preserving cognitive function in old age.

The aim of the present hypothesis-driven study was to examine longitudinally the sex-specific interplay of LS and CR for cognitive decline in aging. We hypothesized that higher levels of LS would be independently associated with a slower decline in cognitive functioning over time and that this association would vary by CR levels and sex.

## Materials and methods

### Study population

In this research we used the data from the Swedish National Study on Aging and Care in Kungsholmen (SNAC-K; http://www.snac-k.se), an ongoing population-based longitudinal study of older adults aged ≥ 60 years, living at home or in an institution in Kungsholmen (Central Stockholm, Sweden) [39]. A total of 3363 individuals took part in the baseline examination between 2001 and 2004, with a total participation rate of 73%. Regular follow-ups (six-year interval for the younger age cohorts [< 78 years at baseline], three-year interval for the older age cohorts [≥ 78 years at baseline]) have since been performed. In this specific study, we use follow-up data from five waves, spanning a maximum of 15 years (See Figure S1). Each wave included a comprehensive assessment comprising nurse interviews, clinical, functional, and psychological assessments, and laboratory testing.

To construct the analytical sample, we excluded 745 individuals with a Mini-Mental State Examination (MMSE) [40] score ≤ 24 at baseline, a baseline diagnosis of dementia, Parkinson´s disease, depression, behavioural disorders (e.g., schizophrenia), or missing data in these exclusion criteria (See Figure S2). After additionally removing 747 individuals with missing data on life satisfaction, our analytical sample comprised 1871 individuals who were followed for up to 15 years. SNAC-K was approved by the Regional Ethical Review Board in Stockholm, and written informed consent was obtained from participants or their proxy.

### Life satisfaction

Life satisfaction was assessed at baseline through a validated self-reported instrument, the Life Satisfaction Index A (LSI-A) [9, 41]. Specifically designed to evaluate well-being in aging, it captures five key components of life satisfaction: zest versus apathy, resolution and fortitude, congruence between desired and achieved goals, positive self-concept, and mood tone. The scale consists of 20 items, each rated as “agree,” “disagree,” or “uncertain” and a high score indicates greater levels of LSI. In the current study, the index was dichotomized according to the median as low (≤ 65) and high (> 65).

### Cognitive reserve

In accordance with previous literature [42, 43], stimulating factors during the life course (i.e., early life education, midlife substantive work complexity, late-life social network and leisure activities), recognized as proxy variables of cognitive reserve [44], were incorporated into an indicator of CR. The importance of these factors within this construct stems from their well-established association with reduced dementia risk [e.g., 45–49] and altered clinical expression of pathological alterations [50, 51], likely underpinned by neural efficiency and adaptability [52]. Importantly, they reflect an active engagement in cognitively enriching life experiences over the life-course, combined together based on a shared variance approach enabled by SEM. While continued conceptual and methodological refinement of the CR model is ongoing [44], our approach represents a well-accepted methodology in the literature.

In this study, education was categorized into seven levels, from unfinished primary to doctoral studies. Occupational complexity in midlife was measured by evaluating participants’ five longest-held jobs, ranking each for substantive complexity, and averaging the rankings across all reported occupations. Leisure activities in late life were assessed based on self-reported participation over the past year in mental (e.g., reading, games), social (e.g., attending events, volunteering), and physical (e.g., gardening, repairing objects) domains. Social network was assessed according to size (e.g., marital status, number of contacts, degree of social interaction) and perceived support (e.g., satisfaction with relationships, sense of belonging). These lifelong indicators were integrated into a composite CR score using the same structural equation modelling (SEM) and parameters established in the original model [42]. In the current study, the CR index (range − 4.25 to 3.46) was dichotomized according to the median as low (≤ 0.08) and high (> 0.08).

### Assessment of cognitive function

Mini-Mental State Examination (MMSE) [40], a composite measure of global cognitive functioning, was administered at each wave. Despite being susceptible to some ceiling effects and psychometric limitations [53], MMSE is the most widely used cognitive screening test, extensively applied across both clinical and non-clinical settings, providing a straightforward, quick and mostly valid evaluation of cognitive functioning [54]. It covers 11 domains including orientation, registration, attention and calculation, recall, naming, repetition, comprehension, writing and construction.

### Covariates

Covariates included age (continuous), sex (male/female), count of chronic conditions (continuous) [55] and functional dependency (continuous; sum of limitations in activities of daily living [ADL] and instrumental activities of daily living [IADL]). Additionally, we considered personality traits (extraversion, neuroticism, openness to experience) and depressive symptoms. Personality was measured through the short Swedish version of the self-reported NEO Five-Factor Inventory (NEO-FFI) [56, 57] and in this study, we specifically used a version with 36 items; each personality trait was then divided into three categories: low, moderate and high. Regarding depressive symptoms, the 10-item Montgomery-Åsberg Depression Rating Scale (MADRS, total range score 0–60; continuous variable) [58] was used, with scores above 9 indicating a clinically relevant level of depressive symptoms. All covariates were assessed at baseline.

### Statistical analysis

Descriptive statistics by sex and LSI were computed using t-test for continuous variables and χ2 test for categorical ones. Baseline characteristics were also compared between eligible participants, who were excluded due to missing data, and the analytical sample (See Table S3). Linear mixed models were used to evaluate the association of LSI and CR (both as time-invariant variables) with changes in global cognitive functioning over 15 years. Models included interactions between the two exposures of interest (i.e., LSI and CR) and follow-up time as fixed effects, alongside random effects for individuals (i.e., intercept) and time (i.e., slope). The interaction terms represent the effect of LSI and CR on the longitudinal rate of cognitive change, as assessed by MMSE, and its positive β-coefficient would indicate slower cognitive decline with higher levels of explanatory factors. Both LSI and CR were operationalized as dichotomized variables based on their medians both to account for potential non-linear associations with the outcome and to ease the interpretation of the findings [9].

Separate models were first run for LSI and CR to assess their individual impact on MMSE, followed by sex-stratified to evaluate association pattern between men and women. Next, both predictors were entered into the same model to estimate their independent effects, alongside sex-stratified sub-models. To explore the moderating effect of CR on the relationship between LSI and cognitive functioning, the models were stratified into two groups, based on low or high level of CR (as well as subsequently by sex, as previously). Stepwise adjustment was used for all analyses: Model 1 adjusted for age and sex [in pooled sex analyses] while Model 2 additionally included count of chronic conditions, functional impairments, personality traits, and depressive symptoms.

## Results

### Baseline characteristics of study participants

The study population consisted of 1871 individuals (mean age = 71.54, SD = 9.56), 59.9% of whom were female. At baseline, participants showed overall well-preserved cognitive functioning and low depressive symptoms (Table 1). Men and women differed significantly on all background factors, except for leisure activities, social network richness, disability, baseline MMSE, and depressive symptoms. Notably, the latter two variables were part of the inclusion criteria, and therefore their ranges were restricted. LSI levels varied across all descriptive characteristics (Table 1), justifying their inclusion in the analytical models.

**Table 1:** Baseline sociodemographic, clinical and lifestyles characteristics of the total study sample and by sex and Life Satisfaction Index

	Total sample (*n* = 1871)		Sex					Life Satisfaction Index (LSI)				
			Men (*n* = 751)		Women (*n* = 1120)		*p* Value	Low (*n* = 1099)		High (*n* = 772)		*p* Value
Age (%)												
≤ 78	1209	(64.6)	519	(69.1)	690	(61.6)	0.001	606	(55.1)	603	(78.1)	< 0.001
> 78	662	(35.4)	232	(30.9)	430	(38.4)		493	(44.9)	169	(21.9)	
Cognitive function (M ± SD)												
MMSE	29.04 ± 1.12		29 ± 1.13		29.07 ± 1.12		0.191	28.91 ± 1.21		29.24 ± 0.98		<0.001
Depressive symptoms (%)												
MADRS ≤ 9	1773	(97)	716	(97.5)	1057	(96.6)	0.316	1013	(95)	760	(99.7)	< 0.001
MADRS > 9	55	(3)	18	(2.5)	37	(3.4)		53	(5)	2	(0.3)	
Chronic conditions (%)												
≤ 1	321	(17.2)	149	(19.8)	172	(15.4)	0.014	151	(13.7)	170	(22)	<0.001
> 1	1550	(82.8)	602	(80.2)	948	(84.6)		948	(86.3)	602	(78)	
Disability (M ± SD)	0.18 ± 0.76		0.16 ± 0.75		0.20 ± 0.77		0.257	0.27 ± 0.96		0.06 ± 0.32		<0.001
Personality: extraversion (%)												
Low	501	(26.9)	238	(31.8)	263	(23.7)	< 0.001	400	(36.8)	101	(13.1)	<0.001
Intermediate	754	(40.5)	294	(39.3)	460	(41.4)		455	(41.8)	299	(38.7)	
High	605	(32.5)	217	(29)	388	(34.9)		233	(21.4)	372	(48.2)	
Personality: neuroticism (%)												
Low	801	(43.1)	385	(51.4)	416	(37.4)	< 0.001	327	(30.1)	474	(61.4)	<0.001
Intermediate	570	(30.6)	196	(26.2)	374	(33.7)		354	(32.5)	216	(28)	
High	489	(26.3)	168	(22.4)	321	(28.9)		407	(37.4)	82	(10.6)	
Personality: openness (%)												
Low	643	(34.6)	300	(40.1)	343	(30.9)	<0.001	453	(41.6)	190	(24.6)	<0.001
Intermediate	557	(29.9)	208	(27.8)	349	(31.4)		326	(30)	231	(29.9)	
High	660	(35.5)	241	(32.2)	419	(37.7)		309	(28.4)	351	(45.5)	
CR (%)												
Low	778	(44.8)	289	(41.4)	489	(47.1)	0.022	599	(59.1)	179	(24.8)	<0.001
High	959	(55.2)	409	(58.6)	550	(52.9)		415	(40.9)	544	(75.2)	
Education (%)												
Elementary	234	(12.5)	85	(11.3)	149	(13.3)	<0.001	180	(16.4)	54	(7)	<0.001
High school	917	(49)	317	(42.2)	600	(53.6)		567	(51.6)	350	(45.3)	
University	720	(38.5)	349	(46.5)	371	(33.1)		352	(32)	368	(47.7)	
Work complexity (%)												
Low	660	(35.9)	157	(21.2)	503	(45.9)	<0.001	452	(42.2)	208	(27.2)	<0.001
Intermediate	678	(36.9)	323	(43.6)	355	(32.4)		368	(34.3)	310	(40.5)	
High	499	(27.2)	260	(35.1)	239	(21.8)		252	(23.5)	247	(32.3)	
Social network (%)												
Low	435	(23.7)	172	(23.4)	263	(23.8)	0.074	360	(33.4)	75	(9.9)	<0.001
Intermediate	663	(36.1)	245	(33.4)	418	(37.9)		410	(38)	253	(33.4)	
High	739	(40.2)	317	(43.2)	422	(38.3)		309	(28.6)	430	(56.7)	
Leisure activities (%)												
Low	487	(27.7)	203	(29)	284	(26.8)	0.607	360	(35.1)	127	(17.3)	<0.001
Intermediate	804	(45.7)	315	(44.9)	489	(46.1)		459	(44.7)	345	(47)	
High	470	(26.7)	183	(26.1)	287	(27.1)		208	(20.3)	262	(35.7)	
LSI (%)												
Low	1099	(58.7)	410	(54.6)	689	(61.5)	0.003					
High	772	(41.3)	341	(45.4)	431	(38.5)						

*M * mean, *SD * standard deviation, *MMSE* Mini-Mental State Examination, *MADRS* Montgomery-Åsberg Depression Rating Scale, *CR* cognitive reserve, *LSI * life satisfaction index

Levels (high/low) of cognitive reserve and life satisfaction index according to the median of the distribution

### Associations of LSI and CR with MMSE decline

Separately, both higher levels of CR and LSI were associated with greater cognitive preservation over the 15-year period (β*time = 0.49 and β*time = 0.33 respectively, both *p* < 0.001). This pattern held across minimally adjusted and fully adjusted models (See Table 2; first column). When CR and LSI were entered into the same model (Table 3), each remained independently associated with cognitive preservation after adjustments (β*time = 0.43 and β = 0.22 respectively, both *p* < 0.001). As presented in Table 4, the association of LSI with cognitive preservation was statistically significant in individuals with both low- (β*time = 0.32; *p* < 0.01, confounder-adjusted) and high CR (β*time = 0.18; *p* < 0.001, confounder-adjusted).

**Table 2:** Associations of CR and LSI levels with MMSE score over the 15-year follow-up for the full sample and stratified by sex

		Sexes combined			Men			Women		
		*n*	β (CI 95%)	*p* Value	*n*	β (CI 95%)	*p* Value	*n*	β (CI 95%)	*p* Value
CR			Reference			Reference			Reference	
Low	Model I	1737	0.48 (0.38; 0.57)	< 0.001	698	0.27 (0.12; 0.42)	<0.001	1039	0.59 (0.46; 0.77)	<0.001
High	Model II	1653	0.49 (0.39; 0.59)	< 0.001	668	0.24 (0.08; 0.39)	0.001	985	0.62 (0.49; 0.75)	< 0.001
LSI			Reference			Reference			Reference	
Low	Model I	1871	0.3 (0.20; 0.39)	< 0.001	751	0.14 (0.001; 0.30)	0.052	1120	0.39 (0.26; 0.52)	< 0.001
High	Model II	1775	0.33 (0.23; 0.43)	< 0.001	716	0.15 (0.01; 0.30)	0.03	1059	0.43 (0.30; 0.56)	< 0.001

Model I: adjusted by sex and age

Model II: adjusted additionally by MADRS (Montgomery-Åsberg Depression Rating Scale), number of chronic pathologies, disability and personality traits. Levels (high/low) of CR and LSI are dichotomized based on the median of the distribution. Positive coefficients refer to lower decline in the MMSE score compared to the reference group

**Table 3:** Associations of CR and LSI with MMSE score over the 15-year follow-up, in the same model, for the full sample and stratified by sex

		Sexes combined			Men			Women		
		*n*	β (CI 95%)	*p* Value	*n*	β (CI 95%)	*p* Value	*n*	β (CI 95%)	*p* Value
CR			Reference			Reference			Reference	
Low	Model I	1737	0.42 (0.32; 0.53)	<0.001	698	0.25 (0.09; 0.41)	0.001	1039	0.52 (0.38; 0.65)	<0.001
High	Model II	1653	0.43 (0.32; 0.53)	< 0.001	668	0.21 (0.05; 0.37)	0.009	985	0.54 (0.40; 0.68)	< 0.001
LSI			Reference			Reference			Reference	
Low	Model I	1737	0.2 (0.09; 0.30)	< 0.001	698	0.08 (-0.07; 0.22)	0.286	1039	0.26 (0.13; 0.39)	< 0.001
High	Model II	1653	0.22 (0.12; 0.33)	< 0.001	668	0.1 (-0.05; 0.25)	0.176	985	0.30 (0.16; 0.43)	< 0.001

Model I: adjusted by sex and age

Model II: adjusted additionally by MADRS (Montgomery-Åsberg Depression Rating Scale), number of chronic pathologies, disability and personality traits. Levels (high/low) of CR and LSI are dichotomized based on the median of the distribution. Positive coefficients refer to lower decline in the MMSE score compared to the reference group

**Table 4:** Association of LSI with MMSE score over the 15-year follow-up, stratified by CR level and by sex

			Sexes combined			Men			Women		
			*n*	β (CI 95%)	*p* Value	*n*	β (CI 95%)	*p* Value	*n*	β (CI 95%)	*p* Value
Low CR											
	LSI			Reference			Reference			Reference	
	Low	Model I	778	0.29 (0.09; 0.49)	0.004	289	0.16 (-0.11; 0.43)	0.238	489	0.34 (0.07; 0.61)	0.012
	High	Model II	731	0.32 (0.11; 0.52)	0.002	274	0.16 (-0.12; 0.44)	0.265	457	0.40 (0.12; 0.67)	0.004
High CR											
	LSI			Reference			Reference			Reference	
	Low	Model I	959	0.15 (0.04; 0.25)	0.005	409	0.04 (-0.13; 0.21)	0.633	550	0.22 (0.09; 0.36)	<0.001
	High	Model II	922	0.18 (0.07; 0.29)	<0.001	394	0.08 (-0.09; 0.26)	0.362	528	0.26 (0.12; 0.40)	<0.001

Model I: adjusted by sex and age

Model II: adjusted additionally by MADRS (Montgomery-Åsberg Depression Rating Scale), number of chronic pathologies, disability and personality traits. Levels (high/low) of CR and LSI are dichotomized based on the median of the distribution. Positive coefficients refer to lower decline in the MMSE score compared to the reference group

### Sex-stratified association of LSI and CR with MMSE decline

Sex-stratified analyses aligned with the full sample results in indicating protective effects of CR and LSI from separate models in both men (β*time_cr_ = 0.24, *p* < 0.01 and β*time_LSI_ = 0.15, *p* < 0.05) and women (β*time_cr_ = 0.62, *p* < 0.001 and β*time_LSI_ = 0.43, *p* < 0.001; see Table 2). However, when both factors were entered into the same model, among men, LSI was no longer associated with cognitive preservation, while CR preserved its protective effect (β*time = 0.21, *p* < 0.01). In contrast, among women, both factors continued being associated with slower MMSE decline in a mutually adjusted model (β*time_cr_ = 0.54, *p* < 0.001 and β*time_LSI_ = 0.30, *p* < 0.001; see Table 3). Examining sex-specific interplay of CR and LSI largely mirrored these findings. In men, LSI was not associated with changes in the MMSE scores in both low and high CR sub-strata. In contrast, protective effects of LSI on cognitive preservation were present among women with both low- (β*time = 0.40, *p* < 0.01) and high CR (β*time = 0.26, *p* < 0.001; see Table 4).

## Discussion

In this population-based longitudinal study, we found that higher psychological well-being, as measured by life satisfaction, was associated with better cognitive preservation over a 15-year period. This relationship showed a sex-specific pattern influenced by cognitive reserve: in women LSI remained associated with cognitive decline across CR levels, whereas in men, incorporating CR rendered LSI’s cognitive effect no longer statistically significant. These findings underline the intricate interplay of psychological wellbeing and cognitive resilience between sexes and could suggest that among women, promoting LSI may represent an additional protection against cognitive decline. In men, although an influence of life satisfaction on cognitive functioning over time cannot be ruled out, particular attention may be given to enriching lifelong CR.

Our findings align with previous studies that investigated the impact of psychological well-being on cognitive outcomes over time, both in terms of general cognitive functioning as well as for specific domains. For instance, in the Berlin Aging Study, higher levels of well-being predicted positive deviations from the expected linear decline in perceptual speed over a two-year period even after adjusting for health constraints, personality, and social participation [59]. Similar evidence from other longitudinal works [19, 60] reported that higher levels of LSI were prospectively associated with a better cognitive function, including a lower risk of incident dementia. In our study, the association of LSI with slower cognitive decline was preserved after accounting for measures of clinical health, functional status and depressive symptomatology. Notably, we also considered personality, which is known to have a long-term influence on both well-being [e.g., 61], and cognitive impairment [62]. LSI’s cognitive benefits may partly act, among others, through cardiovascular health, which contributes to the maintenance of cognitive functioning and protects against dementia [63]. Higher LSI would be also associated with healthier lifestyle behaviours (e.g., more physical activity, less smoking, better sleep and diet) and more adaptive stress responses [22].

We also reported that CR retained its protective association against cognitive decline independent of LSI. CR is a well-established factor in dementia [44], although its role in shaping cognitive decline slopes is more debated [64]. We found CR to be consistently protective of cognitive deterioration, also in mutually adjusted models incorporating LSI, both in both men and women. While MMSE is a well-established tool for screening for cognitive impairment, it has a ceiling effect when measuring higher levels of functioning, underestimating the range of CR’s protective cognitive effects – a bias that can be accentuated when exposure (i.e., CR) is also associated with the levels of the outcome (i.e., cognition) [64].

Importantly, our results demonstrated notable sex differences in the interplay between LSI and CR for cognitive change. When examining each factor’s independent contribution to cognitive decline as measured by the MMSE, both had a protective effect among women, whereas only CR, and not LSI, was associated with cognitive preservation in men. Sex and gender differences in protective factors against cognitive decline remain poorly understood, although recently explicit calls in the literature have been made to expand the understanding of these processes [36, 65]. In a recent systematic review [35], sex differences emerged in risk factors associated with the progression from Mild Cognitive Impairment (MCI) to dementia, including sociodemographic, health, psychological factors, genetics, and biomarker levels. For instance, APOE4 status and depression were found to increase the risk of progression to dementia predominantly in women, whereas brain health markers from MRI and CSF appeared more relevant for men. Even some components of CR itself (e.g., education, occupation, physical activity) appear to be strongly influenced by gender [66, 67].

Specifically focusing on psychological well-being, it is worth considering sex differences in the pathways towards LSI, as these may also have a differential effect on cognitive outcomes. For example, a study of 80-year-old twins from Sweden has found that in women, LSI was closely linked to self-rated physical health and depressive symptoms, whereas in men, widowhood appeared to be the strongest single determinant of LSI [68]. Although speculative, it is possible that the overlap between CR (which also incorporates civil status, social connections and social support) and LSI was greater for men than for women. Conversely, among women, whose well-being may be susceptible also to the effects of depressed mood [e.g., 69, 70], as well as to more frequent multimorbid conditions [71], CR and LSI could be less interconnected.

These results and their interpretation should be considered within the Swedish context. Even though Sweden experienced a gradual increase in education and employment opportunities among individuals born during the first half of the 20th century, especially among women [72] and nowadays is the first in terms of gender equality [73], men likely could have had more opportunities to engage in activities that subsequently contributed to cognitive reserve. Moreover, in this context, higher socioeconomic status, especially educational attainment, seems to have also a positive impact on social participation and linked to more active social profiles in later life [72].

From a practical perspective, our findings could provide a starting point for the design of interventions that, in addition to promoting cognitive functioning in aging, may also make individuals more aware of the importance of psychological well-being; this could also translate, for instance, into psychoeducational interventions, particularly targeting woman. Furthermore, for example, since dementia protective and risk factors are often investigated during clinical assessment for preventive purposes (e.g., in Brain Health Services) [74], the inclusion of specific instruments to assess life satisfaction (and more generally, psychological well-being) could also be suggested.

Overall, further literature is needed explore sex-specific drivers of life satisfaction, particularly as they relate to sex differences in old-age cognitive outcomes. Future studies should also examine whether the sex-specific interactions between CR and LS may differ across different cognitive domains (e.g., executive functions, speed processing, memory), given that previous research reported domain-specific effects of psychological well-being on cognition [60]. In the current study, the use of a global measure of cognitive functioning was justified by the fact that, within the SNAC-K cohort, the longitudinal relationship between LS, CR and cognitive outcomes had not yet been investigated. In addition, it is worth mentioning that LS and CR could also be conceptually and methodologically intertwined, particularly over time, as activity engagement (one of the CR components) has been suggested to be one of the mechanisms through which personality change (and subsequently change in LS) is enacted in late life [34, 75, 76]. Future work leveraging longitudinal assessments of both CR and LS could explore the issue of conceptual overlap in greater detail.

Among the study strengths, we point out the use of a large sample of older adults, followed longitudinally over a 15-year period, and the access to data on several potential confounders such as personality, depressive symptoms, chronic diseases, and functional impairment. The novelty of our study also represents a substantial strength. To our knowledge, this work is among the first to examine longitudinally the interaction between dementia protective factors, such as CR and LS, also considering sex-specific differences in their impact.

Nonetheless, some limitations should be acknowledged. First, a sizeable proportion of participants had missing LSI data (28.6%), which consequently reduced our sample size and impacted the generalizability of our results. The self-report modality of the scale may have led to a higher number of missing data, exacerbated by the fact that the topic may be more challenging given the personal reflection required. However, it could raise concerns about potential selection bias among participants. Second, sex-stratified analyses revealed an imbalance in sex distribution in our sample (40.1% men, 59.9% women), to be considered when interpreting the statistical significance of the estimates. Indeed, the under-representation of men in the sample could have reduced the power for sex-stratified analyses, thus limiting the possibility to detect LS effects in the male group. For this reason, it cannot be completely excluded that life satisfaction may impact cognitive functioning in men as well.

Third, despite adjusting for multiple covariates and excluding participants with dementia and psychiatric disorders, additional confounding variables may still influence the relationship between LSI and cognition. For instance, we lacked information on early-life social environments, which might have contributed to shaping stable individual traits, but also possibly affect cognitive change trajectories in late life. Future studies should consider investigating and including such factors to better understand the complex interplay between psychological well-being and cognitive aging. Potential reverse causality may not be excluded, as lower cognition (at preclinical or subjective stage) could affect life satisfaction, albeit reverse-causation biasing CR is less likely, given their temporal ordering over the life course. In order to mitigate this concern, participants with pronouncedly low baseline MMSE (≤ 24) were excluded, due to the possibility of exposure misclassification as a result of cognitive impairment. Furthermore, adjusting the analysis for multiple covariates may have further reduced, although not eliminated, the risk of reverse causality. This issue, alongside the static (time-invariant) exposures, suggest that our findings should be considered in light of some residual reverse causation.

Finally, it is also worth noting that our results linked to CR are based on participants living in the Kungsholmen district of Stockholm, a very affluent area of the city with high material resources and health literacy. This may limit the generalizability of our results both to other Swedish areas as well as internationally.

## Conclusion

This population-based longitudinal cohort study sought to explore the sex-specific interplay between psychological well-being, as measured by life satisfaction, and cognitive reserve, captured through multiple mentally and socially stimulating activities throughout life. Using data from the SNAC-K, we found that for women, higher levels in life satisfaction may be associated with a better global cognitive functioning over time, whereas for men, enhancing lifelong cognitive reserve would appear more relevant. Overall, our findings suggest the importance of considering sex-specific differences in studying structural (i.e., CR) as well as experiential (i.e., psychological well-being, LSI) protective factors in cognitive aging. Our study may represent a valuable contribution toward the understanding of the role of psychological well-being, alone, or alongside cognitive reserve enrichment, as potential targets for more personalized and sex-sensitive interventions to promote healthy aging.

## Supplementary Information

Supplementary Material 1.

## Data Availability

Data are from the SNAC-K project, a population-based study on aging and dementia (http://www.snac-k.se/). The data collection protocol, including the Mini-Mental State Examination (MMSE), was established in 2000, prior to the introduction of licensing requirements. Access to these original data is available to the research community upon approval by the SNAC-K data management and maintenance committee. Applications for accessing these data can be submitted at [https://www.snac-k.se/for-researchers/application-form/](https:/www.snac-k.se/for-researchers/application-form) .

## Abbreviations

LS: Life satisfaction
CR: Cognitive reserve
MMSE: Mini-Mental State Examination
SNAC-K: Swedish National Study on Aging and Care in Kungsholmen
SEM: Structural equation modelling
ADL: Activities of daily living
IADL: Instrumental activities of daily living
NEO-FFI: NEO Five-Factor Inventory
MADRS: Montgomery-Åsberg Depression Rating Scale
MCI: Mild Cognitive Impairment
