# Supplementary material for: The interplay of life satisfaction and cognitive reserve: implications for cognitive changes in old age
Source: BMC Geriatr. 2026 Mar 28;26:488. doi: 10.1186/s12877-026-07391-0 (PMC13064251; doi:10.1186/s12877-026-07391-0)
Supplement: Supplementary file 1 — Supplementary Material 1. [file 12877_2026_7391_MOESM1_ESM.docx]

SUPPLEMENTARY MATERIAL

**The interplay of life satisfaction and cognitive reserve: implications for cognitive changes in old age**

**Figure S1.** Flowchart of study population.


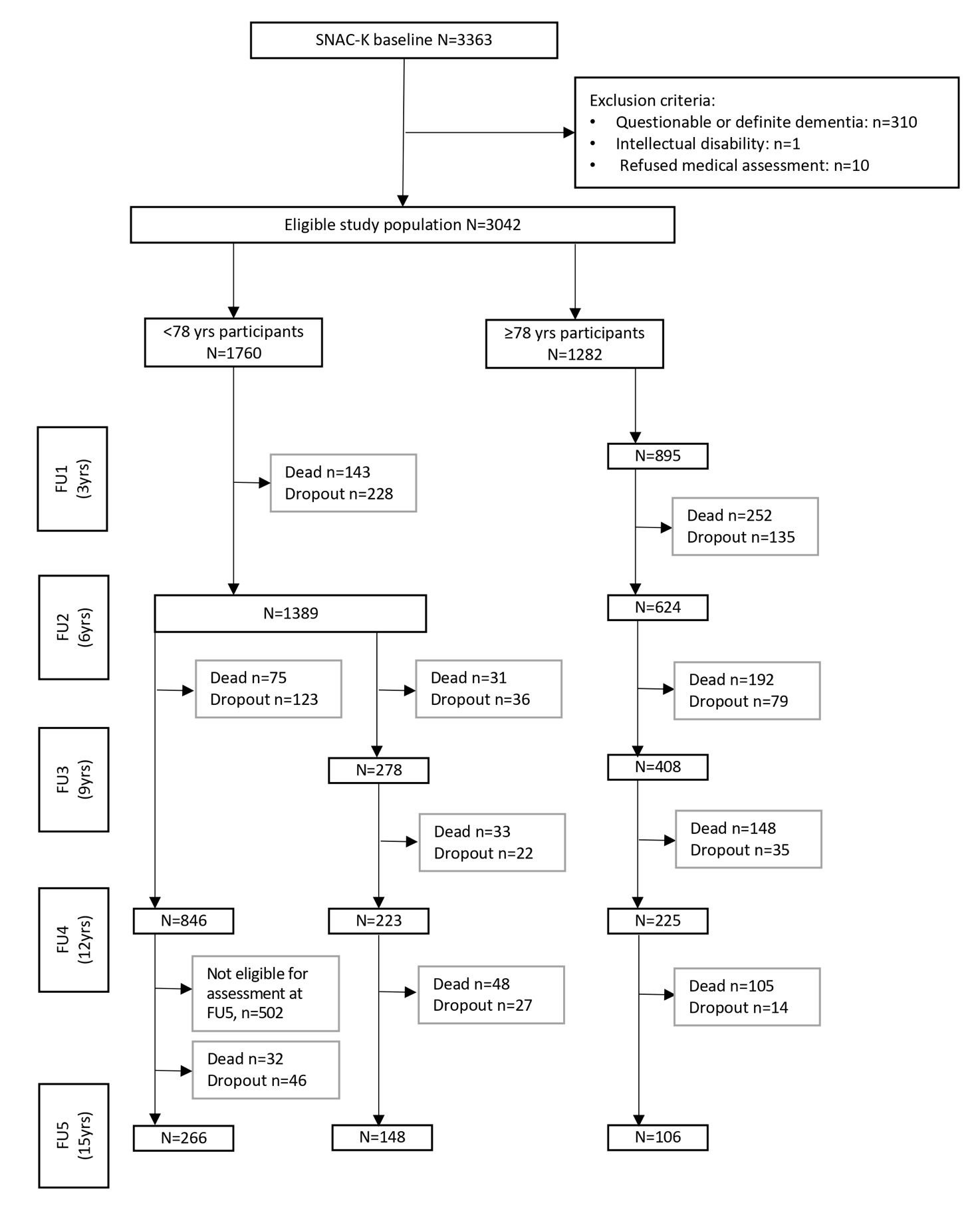


For more information on the study design, we refer to the SNAC-K website (<https://www.snac-k.se/>).

**Figure S2.** Flow diagram of excluded participants and final analytical sample.


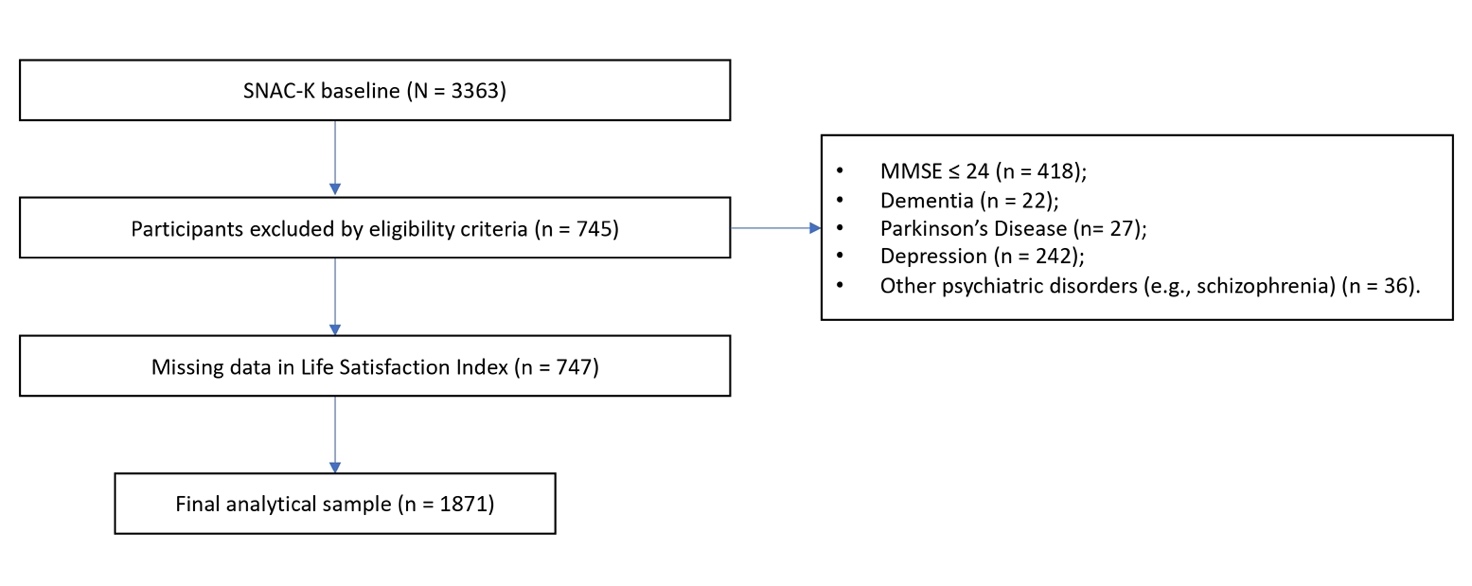


**Table S3.** Baseline sociodemographic, clinical, and lifestyle characteristics of included participants and eligible participants, excluded due to missing data.

|  | **Total sample** | | | | |
| --- | --- | --- | --- | --- | --- |
|  | Included (*n* = 1871) | | Excluded Na (*n* = 747) | | *p* Value |
| **Sex** |  |  |  |  |  |
| Women | 1120 | (59.9) | 496 | (66.4) | 0.002 |
| Men | 751 | (40.1) | 251 | (33.6) |  |
|  |  |  |  |  |  |
| **Age yr** | 71.54 ± 9.56 | | 75.84 ± 11.27 | | <0.001 |
| ≤ 78 | 1209 | (64.6) | 358 | (47.9) | <0.001 |
| > 78 | 662 | (35.4) | 389 | (52.1) |  |
|  |  |  |  |  |  |
| **Mini Mental State Examination (MMSE)** | 29.04 ± 1.12 | | 28.48 ± 1.43 | | <0.001 |
|  |  |  |  |  |  |
| **Depressive symptoms** |  |  |  |  |  |
| MADRS ≤9 | 1773 | (97) | 680 | (95.5) | 0.084 |
| MADRS >9 | 55 | (3) | 32 | (4.5) |  |
|  |  |  |  |  |  |
| **Chronic conditions** |  |  |  |  |  |
| ≤ 1 | 321 | (17.2) | 93 | (12.4) | 0.003 |
| > 1 | 1550 | (82.8) | 654 | (87.6) |  |
|  |  |  |  |  |  |
| **Disability** | 0.18 ± 0.76 | | 0.79 ± 1.81 | | <0.001 |
|  |  |  |  |  |  |
| **Personality: extraversion** |  |  |  |  |  |
| Low | 501 | (26.9) | 80 | (35.6) | 0.01 |
| Intermediate | 754 | (40.5) | 89 | (39.6) |  |
| High | 605 | (32.5) | 56 | (24.9) |  |
| **Personality: neuroticism** |  |  |  |  |  |
| Low | 801 | (43.1) | 77 | (34.2) | 0.014 |
| Intermediate | 570 | (30.6) | 71 | (31.6) |  |
| High | 489 | (26.3) | 77 | (34.2) |  |
| **Personality: openness** |  |  |  |  |  |
| Low | 643 | (34.6) | 79 | (35.1) | 0.619 |
| Intermediate | 557 | (29.9) | 73 | (32.4) |  |
| High | 660 | (35.5) | 73 | (32.4) |  |
|  |  |  |  |  |  |
| **Cognitive reserve (CR)** |  |  |  |  |  |
| Low | 778 | (44.8) | 413 | (64.5) | <0.001 |
| High | 959 | (55.2) | 227 | (35.5) |  |
| **Education** |  |  |  |  |  |
| Elementary | 234 | (12.5) | 147 | (19.8) | <0.001 |
| High school | 917 | (49) | 368 | (49.6) |  |
| University | 720 | (38.5) | 227 | (30.6) |  |
| **Work complexity** |  |  |  |  |  |
| Low | 660 | (35.9) | 288 | (41.4) | 0.022 |
| Intermediate | 678 | (36.9) | 248 | (35.6) |  |
| High | 499 | (27.2) | 160 | (23) |  |
| **Social network** |  |  |  |  |  |
| Low | 435 | (23.7) | 264 | (41.6) | <0.001 |
| Intermediate | 663 | (36.1) | 204 | (32.1) |  |
| High | 739 | (40.2) | 167 | (26.3) |  |
| **Leisure activities** |  |  |  |  |  |
| Low | 487 | (27.7) | 189 | (35.9) | <0.001 |
| Intermediate | 804 | (45.7) | 244 | (46.4) |  |
| High | 470 | (26.7) | 93 | (17.7) |  |
